# Micro-Nanoplastic Exposure and Lung Cancer Biomarkers: The Role of Extracellular Vesicle-Mediated Intercellular Communication

**DOI:** 10.3390/ijms27114887

**Published:** 2026-05-28

**Authors:** Melania Dovizio, Dorian Fink, Marco Gatta, Annalisa Bruno, Cristina Milillo, Francesca D’Ascanio, Faiza Ameen, Piero Di Carlo, Piero Chiacchiaretta, Paola Lanuti, Amedeo Amedei, Patrizia Ballerini

**Affiliations:** 1 Department of Innovative Technologies in Medicine & Dentistry, “G. d’Annunzio” University of Chieti-Pescara, 66100 Chieti, Italy; m.dovizio@unich.it (M.D.); marco.gatta@phd.unich.it (M.G.); cristina.milillo@unich.it (C.M.); faiza.ameen@phd.unich.it (F.A.); piero.dicarlo@unich.it (P.D.C.); p.chiacchiaretta@unich.it (P.C.); patrizia.ballerini@unich.it (P.B.); 2 Center for Advanced Studies and Technology (CAST), “G. d’Annunzio” University of Chieti-Pescara, 66100 Chieti, Italy; francesca.dascanio@unidav.it (F.D.); p.lanuti@unich.it (P.L.); 3 Department of Experimental and Clinical Medicine, University of Florence, 50121 Florence, Italy; dorian.fink@unifi.it (D.F.); amedeo.amedei@unifi.it (A.A.); 4 Department of Humanities, Law and Economics, “Leonardo da Vinci” University, 66010 Torrevecchia Teatina, Italy; 5 Department of Medicine and Aging Sciences, “G. d’Annunzio” University of Chieti-Pescara, 66100 Chieti, Italy

**Keywords:** extracellular vesicles, micro-nanoplastics, lung cancer, biomarkers, gut–lung axis

## Abstract

Micro- and nanoplastics (MNPs) are widespread environmental pollutants, with increasing evidence of human exposure through multiple routes. Their detection in human tissues, including the lungs, raises concerns about their potential impact on respiratory health, including lung cancer (LC). This review synthesizes current evidence on the biological effects of MNP exposure, with a focus on mechanisms potentially relevant to LC. In particular, extracellular vesicles (EVs) are discussed as mediators potentially linking environmental exposure to cellular responses. Experimental studies suggest that MNPs may induce oxidative stress, inflammation, and genotoxicity, and may alter EV biogenesis and cargo, thereby influencing pathways involved in epithelial–mesenchymal transition, angiogenesis, and immune modulation. We also explore the potential contribution of the gut–lung axis, where MNP-induced dysbiosis and intestinal barrier disruption may promote systemic inflammatory responses, with bacterial EVs acting as additional mediators. However, evidence directly linking MNP exposure, EV-mediated signaling, and LC is limited and largely derived from experimental models. Key challenges include the lack of standardized detection methods, insufficient dose–response data, and scarce epidemiological evidence. Integrating exposomic and multi-omic approaches, including EV-omics, lipidomics, and metabolomics, is needed to clarify the relevance of these mechanisms and support the identification of potential biomarkers in human disease.

## 1. Introduction

The term “Microplastics” (MPs) was first introduced by Thompson et al. [1] about twenty years ago to describe microscopic plastic fragments approximately 20 μm in diameter. Today, it is widely referred to as 5 mm or smaller solid plastic particles [2]. Plastic debris smaller than 1 μm is now classified as nanoplastics (NPs), which accumulate, but are too small to be individually identified in complex environmental samples [3]. After two decades, it is evident that micro- and nanoplastics (MNPs) contaminate various environments worldwide. Different sources have been identified, such as textile fibers [4], cosmetic products [5], spillage during transportation of pre-production pellets [6], powders, and flakes used for plastic product manufacturing [3,7]. However, fragmentation of larger plastic particles in the environment is the predominant source, and their resistance to biodegradation at the end of their life cycle may contribute to plastic accumulation [3]. Unfortunately, the rate at which macroplastics break down into MPs remains unknown, as does the rate at which MPs further fragment into NPs, as well as the time required for plastic mineralization. Nevertheless, the latter is significantly slower than the rate at which plastics are accumulating in the environment [8]. A deeper understanding of transformation rates would be crucial for assessing the risks posed by plastic exposure to human health.

In recent years, MPs have been detected in various human tissues, organs, and fluids [3], including blood [9], placenta [10,11], liver and kidneys [12], lung [13], and brain [14], suggesting their ability to traverse the body. In addition, they are expelled from the body through feces [15,16,17], urine [18], and exhalation [19].

Current estimates suggest that between 10 and 40 million tons of MPs are released into the environment each year [3]. These estimations are mainly derived from model-based assessments that integrate data on plastic production, waste management, environmental fragmentation, and transport processes. Therefore, they should be considered uncertain due to variability in input datasets, differences in modeling approaches, and the scarcity of direct, standardized measurement methods, particularly for smaller particle size fractions and across environmental compartments.

According to future projections, MP emissions are expected to double by 2040, and models predict severe environmental damage over the next 70–100 years [20]. However, these scenarios are highly dependent on waste management practices and policy interventions. While current models indicate potentially severe long-term environmental impacts over the coming decades, significant gaps in exposure data pose important limitations for risk assessments and understanding of biological effects.

The consequences of direct, prolonged contact with high concentrations of these pollutants are now widely documented. Chronic MNP exposure has been associated with potential adverse health outcomes, including cancer [21,22,23], endocrine disorders [24], inflammatory diseases [25,26], and metabolic disorders [27]; however, the strength and causality of these associations remain to be fully established. Due to their small size (on the nanometer scale), they may pass through biological membranes and enter the bloodstream, raising concerns about their potential long-term impact on human health.

Concentrations of airborne MNPs vary widely between indoor and outdoor environments, with indoor environments generally dominating in terms of both load and residence time. In several studies, indoor air has been shown to have MP values up to 10 times higher than outdoor air [28]. Plausible daily exposure (fragments + fibers) falls within a still-uncertain but expanding range, with sizes predominantly 1–300 µm (while the sub-µm subgroup is likely underestimated due to analytical limitations) [29]. However, the heterogeneity of measurement units (number of particles per m^3^ vs. mass µg/m^3^) and variability in sampling and analysis protocols make it hard to compare the various studies. In this scenario, the lungs are considered a major target for airborne MNP exposure, thereby reinforcing the need to standardize methods and adopt lung dosimetry models to convert inhaled concentrations into internal doses deposited at the epithelial level, accounting for factors such as aerodynamic diameter, shape, density, and charge.

Several studies have confirmed the presence of MNP in lung tissues [13,30,31]. The impact of various MNPs on respiratory health has been studied using a range of approaches, including in vitro studies with lung-derived cell lines, inhalation exposure studies in rodents, and occupational exposure studies in humans [32]. In vitro studies have demonstrated that the MNP uptake by alveolar macrophages or lung epithelial cells is primarily dependent on MNP size and can trigger pro-inflammatory and oxidative stress responses [33,34,35]. In vivo inhalation studies reveal an influx of immune cells and pro-inflammatory responses [36], underscoring the immune system’s crucial role in responding to environmental pollutants. This interaction with resident lung cells may contribute to pathological processes, such as fibrosis and cancer, when dysregulated [37,38,39]. However, understanding the biological mechanisms involved remains quite limited. The responses observed are often specific to the polymer type, MNP size, and experimental model. This makes it hard to study the mechanisms and downstream effects underlying disease development in subjects with high occupational exposure.

To date, an apparent discrepancy exists between the in vitro studies with MNPs and those associated with the development of asthma, pulmonary fibrosis, or lung cancer (LC) in occupational exposure studies. While it is known that inhaling high concentrations of polymers, such as nylon and polyvinyl chloride, can cause adverse effects on the respiratory system, in vitro studies on these materials are limited, as most experimental investigations focus on polystyrene particles [32]. Furthermore, toxicological in vitro and in vivo studies are typically conducted on spherical, uncontaminated particles. In contrast, MPs in the environment undergo atmospheric aging and may coexist in heterogeneous mixtures, potentially with different toxicological effects. In addition, the concentrations used in many studies are orders of magnitude higher than estimated levels of actual inhalation exposure, thus promoting the analysis of acute responses rather than chronic effects at low doses. A recent rapid systematic review using the Navigation Guide method was conducted to understand the effects of MNP exposure on human health [40]. This method evaluates both human and animal evidence, rates the quality and strength of the evidence, integrates the findings, and provides a comprehensive statement on the health effects of MNPs. The analysis by Chartres et al. [40] concluded that MNPs are “suspected” to harm the human reproductive, digestive, and respiratory systems, with a potential link between colon and lung cancer, underscoring the need to clarify the underlying mechanisms of these harmful effects.

Owing to their small size and ability to interact with biological systems, MPs and, in detail, NPs can affect cellular processes, including extracellular vesicle (EV) generation. EVs are double-layered lipid vesicles secreted by cells and are recognized as important mediators of intercellular communication by transporting many metabolites, including proteins, lipids, mRNA, and small non-coding RNAs, which can modulate gene expression (microRNAs). When interacting with recipient cells, these mediators can influence various biological processes, including immunity, inflammation, and tissue repair [41,42]. Recent findings suggest that MNPs may alter EV-mediated cell communication pathways, potentially exacerbating health problems associated with MNP exposure [43,44,45]. Evidence in this field remains limited, but emerging findings suggest that MNPs may influence EV biogenesis and release, thereby complicating the biological effects of MNP exposure. A better understanding of these interactions may help clarify the health impacts of MNP contamination. Beyond interfering with EV-mediated intercellular communication, MNPs may also affect inter-tissue signaling networks, potentially linking environmental exposure to systemic effects. Recent studies have shown that MNPs can damage the intestinal epithelial barrier and activate immune and inflammatory responses [46]. These events may favor the proliferation of pro-inflammatory and opportunistic bacterial populations, decreasing beneficial symbiotic species. At the same time, MNP-induced epithelial damage alters the intestinal microbiota’s ecological niche, thereby modifying oxygen availability and compromising its growth, reproduction, and metabolic activity. Furthermore, MNPs can interfere with the host’s endocrine system, leading to hormonal imbalances that exacerbate gut microbiome (GM) alterations, resulting in intestinal dysbiosis [47].

Among the extraintestinal organs interconnected through the GM axis, there is an anatomical and functional relationship between the gut and the lung, known as the gut–lung axis. Specifically, intestinal dysbiosis and increased mucosal permeability induced by MNPs may promote the translocation of microbial metabolites, inflammatory mediators, and bacterial extracellular vesicles (bEVs) into circulation. When they reach the lungs, these circulating components may contribute to oxidative stress, immune dysregulation, and a pro-tumorigenic microenvironment, thus suggesting a potential mechanistic link between gut disturbances and lung carcinogenesis.

We conducted a narrative review of the literature to provide a comprehensive overview of the evidence supporting a link between MNP exposure and lung carcinogenesis, with a specific emphasis on the molecular mechanisms underlying MNP-induced cellular dysregulation. Furthermore, it aims to discuss emerging insights on the role of EVs as mediators of intercellular communication and potential modulators of MNP toxicity, as well as into the involvement of the gut–lung axis and bEVs in shaping systemic inflammatory and oncogenic responses. The integration of toxicological, cellular, and environmental evidence may contribute to a better understanding of MNP–EV interactions that potentially contribute to LC development and progression and support the identification of potential biomarkers and therapeutic targets for future precision medicine strategies to prevent and manage environmentally induced LC.

## 2. Methods

The literature was examined from January 2004, when the term “microplastic” was first used by Thompson et al. [1], to February 2026, with a focus on studies investigating MNPs in relation to LC. Studies considered eligible were original peer-reviewed articles, reviews, and editorials addressing the presence and biological effects of MNPs, mechanisms potentially involved in carcinogenesis (e.g., inflammation, oxidative stress, and cellular signaling), and the role of EVs or gut–lung axis interactions. Conference abstracts and studies not directly relevant to these topics were excluded.

The search strategy combined broad and targeted queries in PubMed/MEDLINE and Web of Science. An initial extensive search was performed using the terms (“microplastics” OR “nanoplastics”) AND (“lung” OR “respiratory system” OR “pulmonary”), to collect studies addressing the presence and effects of MNPs in the respiratory system. This was integrated by more specific searches focusing on LC (“lung cancer” OR “lung neoplasms”) and on mechanistic pathways, including EV-mediated signaling (“extracellular vesicles” OR “exosomes”) and gut–lung axis interactions (“gut–lung axis” OR “microbiota” OR “dysbiosis”).

The results from these searches were combined, yielding a total of 244 records. After duplicate removal and screening based on title and abstract, performed by two authors, 206 articles were selected for full-text evaluation, and those not meeting the inclusion criteria were excluded. Moreover, the reference lists of relevant articles were screened to identify additional studies.

A total of 164 studies were included in the final analysis. This number includes studies selected through the structured literature search and screening. Then, additional references were included to provide background information and support the review’s contextual framework.

## 3. MNPs: Detection, Analysis, and Toxicological Implications

MNPs are increasingly recognized as environmental contaminants with potential implications for human health, including emerging concerns regarding their role in LC-related processes. The most prevalent MNPs in the environment comprise various polymers produced at different rates in response to global plastic demand. In freshwater and drinking water, the most frequently found polymers are polyethylene (PE), polypropylene (PP), polystyrene (PS), polyvinyl chloride (PVC), and polyethylene terephthalate (PET) [26]. In addition to polymers, various additives are incorporated to improve plastic properties, such as color, flexibility, stability, water resistance, flame retardancy, and UV protection. Many of these additives, including bisphenols, phthalates, per- and polyfluoroalkyl substances, and brominated and organophosphate flame retardants, have been associated with potential health risks [32].

**Figure 1:**
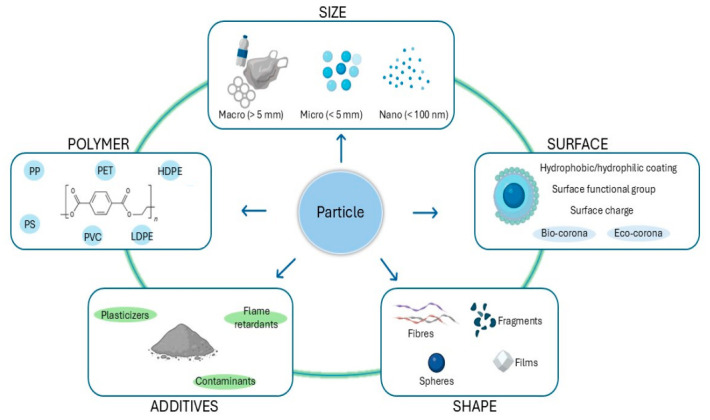
Key metrics of micro- and nanoplastics (MNP) exposure. This figure summarizes the factors that determine interactions with biological systems and influence the bioavailability and bioaccessibility of MNPs. They include exposure levels, particle size and shape, polymer type [i.e., polypropylene (PP), polystyrene (PS), polyvinyl chloride (PVC), and polyethylene terephthalate (PET), high-density polyethylene (HDPE), low-density polyethylene (LDPE)], surface features (for example, surface charge, functional groups), and associated chemicals. In the environment and within the human body, MNPs can also interact with natural organic substances and biomolecules. This interaction leads to the formation of surface crowns, altering their physicochemical properties and affecting their fate, distribution, absorption, transport, biotransformation, and toxic effects.

The toxicological assessment of MNPs involves quantifying exposure and evaluating potential health impacts. Dose metrics mainly include exposure concentration, particle size and shape, polymer identity, and chemical composition (Figure 1) [48]. These factors are thought to influence MNP interactions with biological systems, affecting bioavailability and bioaccessibility [3,48]. Epidemiological research focuses on biological endpoints, including inflammation, oxidative stress, immune responses, and genotoxicity, which may be dose-dependent and can be influenced by the physicochemical properties of MNPs [3,48].

In addition to toxicological and dosimetric aspects, the atmospheric behavior of MNPs is crucial for understanding human exposure. Physical properties such as aerodynamic diameter, density, surface charge, and hygroscopicity influence the transport, persistence, dispersion, and inhalation of airborne particles [49]. Both primary sources (e.g., tire wear, textile fibers, industrial emissions) and secondary sources (fragmentation and resuspension) contribute to airborne MNPs, while dry and wet deposition regulate their concentration at ground level [49]. Emerging evidence also suggests that MNPs may affect atmospheric radiative and cloud formation processes by altering aerosol optical properties [49].

**Figure 2:**
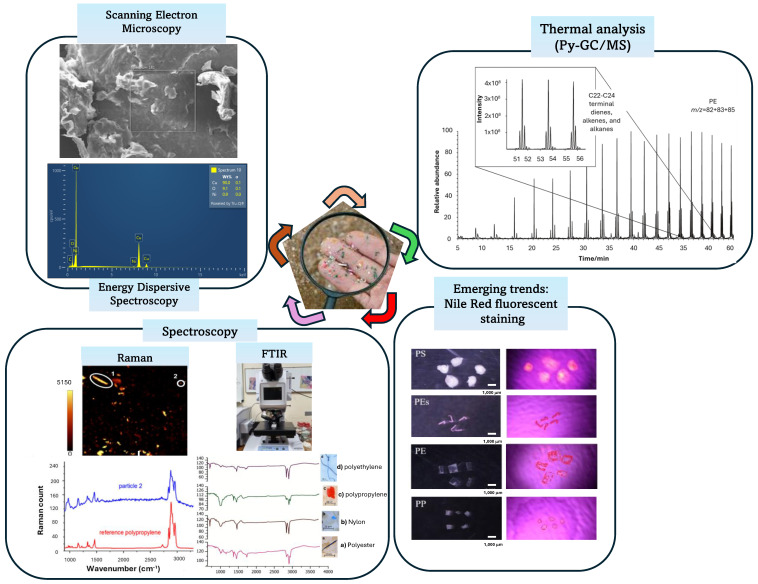
Representative images of physical and chemical techniques for micro- and nanoplastics (MNPs) analysis. Analysis of MNPs primarily involves assessing morphology and topology via microscopy and chemical composition via spectroscopy.

In both the environment and the human body, MNPs can interact with natural organic matter and biomolecules, forming corona-based complexes on their surfaces (Figure 1). Due to this complexity, standardized analytical and monitoring approaches based on microscopy and spectroscopy are urgently needed to generate consistent and comparable data [3,50]. Typically, MNP analysis involves physical and chemical techniques that assess morphology/topology and chemical composition, respectively, using microscopy and spectroscopy [26] (Figure 2). However, comparisons of methods for MNP analysis across studies should be interpreted with caution, given variability in methodologies and units of measurement, as well as the specific limitations of each approach. Moreover, some methods may not fully characterize individual particles, so that the reported quantities could refer to both MPs and NPs.

## 4. MNP–EV Interactions: Implications for Human Health

### 4.1. Definition, Types, and Analysis of Extracellular Vesicles

In the context of MNP exposure, EVs have emerged as potential mediators linking environmental stimuli to cellular responses, including processes relevant to LC development. EVs are a heterogeneous population of membranous structures that have been recognized as mediators of intercellular communication over short or long distances upon release into the extracellular environment [51]. Despite their different origin, EVs generally share a similar biochemical composition, often reflecting the lipid bilayer of the plasma membrane for eukaryotic cell-derived EVs [52] or the structural characteristics of the bacterial cell envelope for bEVs [53].

**Figure 3:**
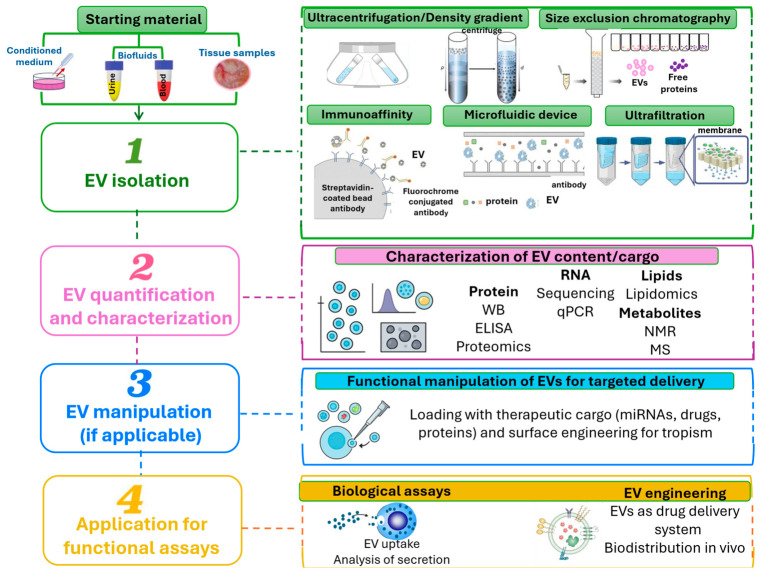
Experimental workflows involving extracellular vesicles (EVs). Phases of EV experimental workflow typically include: (i) isolation, (ii) characterization (i.e., the assessment of concentration, size distribution, surface markers, and molecular content), and (iii) manipulation, when applicable (i.e, loading with specific therapeutic cargoes for targeted delivery). Functional assays for EVs are typically used to assess their bioactivity, quality, and potential as an innovative therapeutic strategy, for example, in cancer therapy and regenerative medicine, as a drug delivery system.

Eukaryotic EVs are conventionally classified by biogenesis into exosomes (40–120 nm), microvesicles (50–1000 nm), and apoptotic bodies (500–2000 nm) [54,55]. Different EV subtypes often exhibit overlapping sizes and phenotypic features; thus, classification based on biogenesis can be challenging and, in some cases, potentially misleading [56]. According to current scientific consensus, experimental workflows involving EVs generally include three central steps: (i) isolation, (ii) characterization (including the assessment of concentration, size distribution, surface markers, and molecular content), and (iii) manipulation, when applicable (i.e, loading with experimental or therapeutic cargoes for targeted delivery) (Figure 3).

Several methodological approaches have been developed for EV isolation. Each has distinct advantages in processing time, cost, purity, and yield. As of today, several primary methods are commonly employed, and their key features are summarized in Table 1.

Ultracentrifugation is one of the most widely used methods, but it is associated with low recovery rates (5–25%) and long centrifugation times. Density gradient centrifugation improves yield up to 60% but still requires an ultracentrifuge. Alternative approaches, such as polymer-based precipitation and size exclusion chromatography (SEC), offer a balance between efficiency (50–70%) and ease of use. Immunoaffinity-based methods and microfluidic technologies allow for high recovery rates (up to 80%) within short timeframes, making them particularly suitable for small-scale or high-sensitivity applications. Lastly, membrane affinity methods provide a very rapid protocol (30 min), though recovery rates are not clearly defined and may require further validation. The cited references offer detailed insights into each method’s protocol and application context.

Morphological characterization of EVs is commonly used to verify their typical cup-shaped structure. This analysis can be performed using high-resolution imaging techniques such as transmission electron microscopy (TEM), cryo-electron microscopy (cryo-EM), and atomic force microscopy (AFM), while complementary approaches including nanoparticle tracking analysis (NTA), dynamic light scattering (DLS), tunable resistive pulse sensing (TRPS), and flow cytometry provide additional information on vesicle size and distribution [54,56]. Once secreted, EVs can act as carriers of a broad spectrum of bioactive molecules, including proteins, lipids, and nucleic acids, potentially affecting the behavior of recipient cells. This cargo delivery system underlies the EV role as mediators of intercellular communication, potentially contributing to local and systemic regulation of key physiological and pathological processes, including cell proliferation, migration, differentiation, apoptosis, immune modulation, inflammation, angiogenesis, fibrosis, and tissue repair [66,67,68,69].

Most cell types have been reported to secrete EVs under both physiological and pathological conditions. EVs have been detected in a wide variety of biological fluids, including, but not limited to, blood, urine, and breast milk, where they can be detected at relatively high concentrations [70,71].

Due to their role in mediating cellular responses to environmental stimuli, EVs are increasingly recognized as potential intermediaries in the biological effects of emerging pollutants, including MNPs.

### 4.2. Mechanisms of MNP–EV Interactions

The scientific interest in EVs is associated with both human and animal diseases. However, their link with environmental pollution represents a growing area of research. EVs’ response to environmental stress was first described in 2017 [72]. Then, most studies focused on EVs as carriers of cellular proteins and RNAs, and on changes in these cargo molecules under environmental stress, as potential mechanisms for disrupting intercellular communication. However, more recent evidence suggests that EVs might also have a protective role against harmful environmental substances [25]. Among them, results from an in vitro study showed that, in response to an acidifying environment, hypoxia, radiation, or cytotoxic drugs, cells release EVs enriched with molecules that affect immune responses and cell growth, or that make the microenvironment more suitable for their survival [73,74]. In vitro and in vivo studies investigating the adverse pulmonary effects of PM2.5 have shown that EVs released from adipose mesenchymal stem cells (ADSCs) exert protective effects by ROS levels and the production of inflammatory cytokines [75]. In addition, PM2.5-induced upregulation of profibrotic signaling pathways, including TGF-β1 and its receptors (TGF-βRI and TGF-βRII), was attenuated following treatment with ADSC-derived EVs. This effect has been associated, at least in part, with the transfer of miRNAs (such as let-7d-5p), modulating the expression of fibrosis-related genes [76].

Thus, taken together, available evidence suggests that EVs may play a role in the interactions between organisms and their environments: they have been proposed to facilitate the transport of vital substances, mediate intra- and intercellular communication, and support organisms’ responses and adaptation to environmental stressors. However, their overall impact remains to be fully elucidated.

The interest in the mechanisms by which widespread pollutants, including PM2.5, cigarette smoke, and ozone, are associated with several clinical settings is fueling an emerging research area. In this scenario, EVs derived from environmental pollutant exposure are increasingly investigated as potential mediators that warrant further exploration [77]. Although only a few studies have examined MNP–EV interactions, these studies suggest that they may be associated with distinct outcomes [25].

In EVs isolated from serum samples of pigs exposed to PET-MPs, 27 miRNAs with altered expression patterns have been identified; some of them have been associated with lifestyle-related diseases (i.e., obesity, insulin resistance, diabetes, and metabolic syndrome) [45]. In addition, the exposure to PET-MPs altered miRNAs associated with cardiovascular disease. In detail, the downregulation of miR-136-3p has been reported to be involved in the regulation of myocardial oxidative stress and inflammation. Expression changes of miRNAs, which can be implicated in cancer development, were also described. Among them, ssc-miR-31 was upregulated at a low dose of PET-MPs [45]. The expression of this miRNA is increased in several cancers, such as non-small cell lung, colorectal, pancreatic, and cervical cancers, and decreased in others, for example, breast, ovarian, prostate, hepatocellular, and gastric cancers [78].

Wang et al. [79] showed that PS-MP exposure in mice was associated with renal MP accumulation, increased release of EVs with an altered ROS-induced profile potentially linked to pro-fibrotic signaling. In human tubular cells, exposure to PS-MPs increased EV production and induced expression of endoplasmic reticulum (ER) stress-related proteins. In fibroblast cells, incubation with conditioned medium from PS-MPs and their cellular uptake induced ROS production and the expression of ER stress-related proteins. Moreover, the expression of fibrosis-related proteins was increased in fibroblasts treated with the conditioned medium from PS-MPs. Altogether, these findings, primarily derived from experimental models, suggest that PS-MPs may be taken up and released via EVs during ROS-associated cellular stress [79].

The MNP accumulation in the gastrointestinal tract may exacerbate intestinal barrier damage, especially in high-risk individuals, such as patients with inflammatory bowel disease, and in conditions of epithelial injury related to lifestyle or aging. Under these conditions, plastic particle penetration through the mucus and epithelial layers is promoted, which may trigger oxidative stress, inflammation, immune dysfunction, reduced cell proliferation, and tissue degeneration. Disruption of the colonic mucus barrier may also enhance MNP translocation into the bloodstream, potentially contributing to systemic toxicity affecting the cardiovascular system and distant organs, including the lungs [26]. In addition, recent evidence suggests a possible association between MNP exposure and colorectal cancer (CRC) [80]. Proposed mechanisms include platelet activation and the release of platelet-derived EVs, carrying lipid mediators, cytokines, growth factors, proteases, and miRNAs that may promote a pro-inflammatory microenvironment supportive of tumorigenesis [26,81,82,83,84,85].

These results suggest that EV cargo and its biological impact on recipient cells can be affected by the presence of MNPs. Firstly, EVs may mediate MNP transport between cells, potentially modulating their biological effects on surrounding cells. On the other hand, extracellular release via EVs may represent a possible mechanism for cellular elimination of MNPs. In this context, MNP-induced EVs may exert heterogeneous effects, potentially contributing to both harmful (e.g., inflammation, tumor-promoting signaling) and adaptive or protective responses, depending on the biological context and exposure conditions. However, evidence on MNP–EV interactions remain limited and largely relies on experimental models.

## 5. MNPs and Lung Carcinogenesis: Current Evidence and Proposed Mechanisms

### 5.1. Pulmonary Effects and Carcinogenic Potential of MNPs

LC, the second most common cancer and the leading cause of cancer-related deaths worldwide, is now recognized as a significant global health concern. It accounts for over 2.2 million new cases and 1.8 million deaths for the year [86]. Smoking remains the leading risk factor, responsible for two-thirds of all LC-related deaths [86]. However, about one-third of LC cases occur in individuals who have never smoked, and its incidence is increasing over time [87]. Among nonsmokers, LC ranks as the fifth leading cause of cancer-related death [87].

Recent evidence identifies air pollution, especially fine particulate matter (PM2.5), with a diameter of ≤2.5 μm, as a significant risk factor for LC in nonsmokers (LCNS) [88] and a contributing factor in the progression of LC, and may be associated with the activation of driver mutations, such as those of the epidermal growth factor receptor (EGFR) [89], and resistance to treatment [90]. Accordingly, several epidemiological studies have shown that LCNS exhibit higher levels of PM2.5 exposure compared to smokers [19,91,92,93].

Despite evidence that PM2.5 may be involved in LC initiation, selection, and/or promotion, the underlying mechanism remains poorly understood. Several lines of evidence suggest that PM2.5 may induce a chronic inflammatory state characterized by a microenvironment that favors DNA damage, cell proliferation, and resistance to apoptosis, potentially contributing to tumorigenesis. Jin et al. [94] reported that lung inflammation induced by PM2.5 is linked to increased levels of reactive oxygen species (ROS), which have been shown to activate pathways such as the EGF-EGFR-protein kinase B (AKT)-nuclear factor kappa B (NF-κB) cascade, interleukin (IL)-1β, and IL-18 [94]. Increased IL-1β release by macrophages has been associated with EGFR mutations, which may contribute to oncogene activation, tumor progression, cancer metastasis, drug resistance, and, finally, LC development, leading to a progenitor-like state in alveolar type II cells mutant for EGFR [93]. Moreover, inflammatory cytokines, such as IL-1β and IL-18, also sustain chronic inflammation, favoring tumor progression. Indeed, PM2.5 exposure has been shown to induce aryl hydrocarbon receptor (AhR)-mediated transcription of transmembrane serine protease 2 (TMPRSS2) and IL-18, thereby potentially contributing to cancer progression in EGFR-mutant cells [95]. AhR activates EGFR through both traditional genomic signaling and non-genomic pathways, promoting cancer cell proliferation and resistance to EGFR-TKIs by stimulating Src tyrosine-protein kinase signaling [96]. Additionally, PM2.5 exposure has been shown to activate the Src/STAT3 pathway, thereby increasing vascular endothelial growth factor (VEGF) production and further exacerbating chronic airway inflammation [97,98].

Although MNPs share several physicochemical properties with PM2.5, including small size, inhalability, and the ability to induce oxidative stress and inflammation, direct evidence linking MNP exposure to LC remains limited. Nevertheless, epidemiological and mechanistic studies on PM2.5 provide a useful framework for hypothesizing potential MNP-related effects. Recent studies have increasingly identified MNPs as components of atmospheric PM2.5 in both indoor and outdoor environments [99].

The WHO recently estimated that humans may inhale up to 3000 airborne MNPs daily and highlighted their potential impact on lung health [100]. MPs have been detected in human lung tissue [13,39,40], bronchoalveolar lavage [101,102], ground-glass nodules [103], and various tumor types. LC showed the highest proportion of MPs, with relatively high PS levels (122.30 ± 154.88 ng/g) as the predominant MP polymer type [104]. Several studies suggest that MNP has harmful effects upon inhalation, mainly on alveolar macrophages and lung epithelial cells [33,34]. MNP exposure has been shown to affect lung epithelial cells, with reported effects including impaired barrier integrity, induction of inflammation, apoptosis, and senescence, and ROS production [105,106,107]. Evidence from primary epithelial and alveolar epithelial models indicates that exposure to MNPs leads to decreased cell viability, metabolic impairment, and long-term lung damage, largely driven by oxidative stress [108,109,110]. The extent of these effects is affected by particle size, concentration, and material [111]. Animal studies support these effects, with PS-NPs reaching the lungs [112] and causing inflammation, macrophage aggregation, and alveolar damage [36,39,113]. Recently, intratracheal instillation of MPs has been shown to induce pro-inflammatory responses and fibrosis in a size- and dose-dependent manner [38]. These studies suggest that MP exposure may contribute to inflammation through p38-mediated NF-κB signaling triggered by mitochondrial injury in the respiratory system [113]. Oxidative stress and alveolar epithelial cell damage have been reported to activate the Wnt/β-catenin signaling pathway [38].

### 5.2. Gut Microbiome and Bacterial EVs in MNP Exposure: Emerging Links to Lung Cancer

In experimental animal models, orally ingested MNPs cross the intestinal barrier and enter systemic circulation [114,115,116]. Humans are continuously exposed to MNPs through food intake; however, although their distribution to multiple organs has been suggested, including the lungs [3,13], direct evidence of organ-specific effects following oral exposure remains limited. In this regard, a recent study [117] has reported that lung inflammation occurs after repeated oral MP administration. While the underlying mechanisms should be further elucidated, these findings support the idea that the systemic effects could occur after gastrointestinal exposure, without directly demonstrating translocation to the lung. Current evidence highlights potential biological effects of MNP exposure, including carcinogenesis-related processes, including tumor-promoting inflammation, oxidative stress, metabolic alterations, genotoxicity, cytotoxicity, and the acquisition of invasive cellular phenotypes [118].

Among the proposed mechanisms, MNP exposure has been associated with changes in the local gut microbiome (GM) and impairment of intestinal barrier integrity, potentially contributing to systemic inflammatory responses. In particular, MNP exposure has been shown to induce gut dysbiosis and lung injury and to be associated with microbiota-derived lactate accumulation. Lactate activates the HIF1α/PTBP1 pathway and promotes cancer progression through the induction of epithelial–mesenchymal transition (EMT) [119]. Similarly, PET-NP exposure altered both the colon and lung microbiota compositions, supporting a possible role of the gut–lung axis in NP-induced toxicity and suggesting that microbial dysbiosis is associated with respiratory health effects [120].

**Figure 4:**
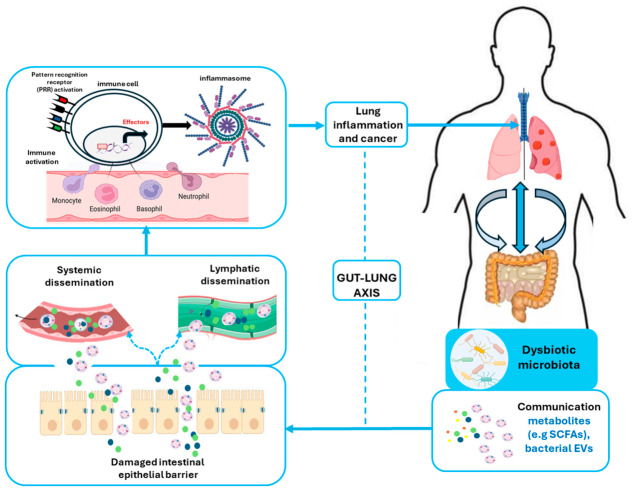
Bacterial extracellular vesicles (bEVs) drive inflammation by activating pattern recognition receptors (PRRs) and the inflammasome, triggering both local and systemic immune responses through the gut–lung axis. Dysbiosis-induced barrier disruption enables translocation of bacteria and their EVs, allowing microbial cargoes to modulate inflammation, tumorigenesis, and immune responses in distant organs. Despite growing evidence linking bEVs to inflammatory conditions, the mechanisms by which bEVs influence cancer through bacterial metabolites, such as short-chain fatty acids (SCFAs), or bioactive cargo remain to be fully elucidated.

In this context, bacterial extracellular vesicles (bEVs) have been proposed as possible mediators of gut–lung communication, because they can transport bioactive microbial molecules and modulate inflammatory and immune responses in distant organs [121]. Several studies suggested the involvement of bEVs in inflammatory diseases, including sepsis, gastrointestinal disorders, periodontal disease, and skin and lung inflammation [122,123,124,125,126]. Moreover, emerging evidence suggests that systemically circulating bEVs may affect various host tissues, as demonstrated in mouse models using bEV-labeling strategies to track their biodistribution after parenteral administration [127,128]. Dysbiosis-associated impairment of epithelial tight junctions may facilitate the translocation of bacteria and their EVs, potentially contributing to tumor-promoting inflammation and immune modulation in distal tissues [129,130] (Figure 4).

Overall, MNP-induced dysbiosis and GM-derived EV signaling represent a biologically plausible mechanistic framework potentially linking environmental exposure to inflammatory and protumorigenic processes; however, direct experimental evidence demonstrating a causal role of bEVs in MNP-associated LC is currently lacking.

## 6. Extracellular Vesicles in Lung Cancer Development and Progression

### 6.1. Contribution of Lung Endothelium and Other Cells to Extracellular Vesicle Circulation

The pulmonary endothelium is considered an important player in the biogenesis and systemic dissemination of EVs, acting both as a source and a target of these vesicular messengers. Endothelial cells (ECs) of the pulmonary vasculature network are dynamic and highly responsive to physiological and pathological stimuli. Their capacity to release EVs, including exosomes and microvesicles, is regulated by environmental cues, such as hypoxia, inflammation, shear stress, and exposure to toxicants, such as cigarette smoke [131,132]. These EVs encapsulate a wide array of bioactive molecules, including cytokines/chemokines and damage-associated molecular patterns (DAMPs), caspases and tissue factor, lipid mediators and nucleic acids, such as miRNAs, reflecting, at least in part, both the state and identity of their native cell. Remarkably, lung EC-derived EVs (EC-EVs) have been reported to exert direct effects on resident innate immune cells, which may contribute to their activation and to inflammatory responses [133]. Moreover, EC-EVs can enter the bloodstream and may influence the function of distant tissues, suggesting their role in systemic intercellular communication, as demonstrated in murine models [134].

Furthermore, Brocco et al. [135] performed a comprehensive phenotypic and proteomic characterization of blood-circulating EVs in patients with advanced NSCLC undergoing immune checkpoint inhibitor therapy. Notably, low levels of endothelial-derived EVs correlated with improved survival in responders, while proteomic profiling identified distinct protein signatures that distinguished responders from non-responders, highlighting the potential of EVs as candidate predictive biomarkers and possible modulators of antitumor immunity [135].

Lung ECs are largely heterogeneous, with macrovascular and microvascular subsets differing in morphology, receptor expression, and vesiculation behavior. This diversity is mirrored in their EV output. For example, pulmonary microvascular ECs are more prone to vesiculation than their macrovascular counterparts upon inflammatory or apoptotic stimulation, likely due to differences in stress sensitivity or activation thresholds [132]. Additionally, the molecular cargo of EC-EVs is highly related to the stimulus: pro-inflammatory cytokines, such as tumor necrosis factor (TNF)-α or injurious agents like lipopolysaccharide (LPS), have been reported to induce the selective packaging of miRNAs (e.g., miR-126, miR-221), adhesion molecules (e.g., ICAM-1, VCAM-1), and procoagulant proteins (e.g., tissue factor), which may collectively shape endothelial–leukocyte interactions, coagulation cascades, and vascular remodeling [136,137].

Beyond ECs, other pulmonary resident and circulating cells contribute to the EV milieu within the lung and systemic compartments. Among them, alveolar epithelial cells can release EVs in response to microbial stimuli [138], while macrophage-derived EVs propagate inflammatory signals or modulate endothelial barrier integrity [133].

The dissemination of lung-derived EVs into the bloodstream suggests their potential as systemic biomarkers for pulmonary endothelial dysfunction. Indeed, circulating EC-EVs are elevated in a range of lung diseases, including acute respiratory distress syndrome (ARDS), chronic obstructive pulmonary disease (COPD), and pulmonary hypertension [132]. These vesicles not only serve as indicators of disease severity but may also participate in disease pathogenesis, for instance, by enhancing vascular permeability or promoting immune cell recruitment. On the other hand, quiescent or progenitor ECs release EVs that may exert protective, anti-inflammatory, or reparative functions, underscoring the dualistic nature of EC-EVs, which depend on the cellular context and microenvironmental cues [139].

In summary, lung endothelial cells, as well as other pulmonary cells, contribute to the generation and blood circulation of EVs. These vesicles may act as versatile conveyors of pulmonary status, modulating distant cellular responses and serving as both sentinels and effectors in lung homeostasis and disease.

### 6.2. Role of Extracellular Vesicles in Cancer Cell Communication

EVs have are increasingly recognized as important mediators of intercellular crosstalk within the tumor microenvironment (TME) and beyond, orchestrating complex signaling networks that may contribute to cancer development, progression, and therapeutic resistance. Tumor-derived EV cargo contains a wide range of mediators, such as proteins, nucleic acids, lipids, and metabolites. Altogether, these components may reflect a molecular signature of the malignant phenotype of the releasing cells. These EVs have been shown to influence the behavior of neighboring stromal and immune cells and have been implicated in the reprogramming of distant tissues to create pre-metastatic niches, as well as in the modulation of antitumor immunity, thereby potentially contributing to tumor evolution rather than merely being products of cellular activity [140].

Cancer cells exploit EV biogenesis pathways to enhance vesicle secretion and selectively package oncogenic molecules [141,142]. Oncogenic mutations such as those in *KRAS*, *MYC*, and *TP53* influence both the quantity and content of EVs; this could promote the dissemination of pro-tumorigenic factors, including some miRNAs (e.g., miR-21, miR-378e), lncRNAs (long-non-coding RNAs), and even double-stranded DNA [143]. These vesicular components contribute to EMT, immune evasion, angiogenesis, and therapy resistance [144,145,146]. Notably, EVs from aggressive tumors often carry immune checkpoint molecules, such as PD-L1, which have been shown to suppress T cell activity and may attenuate responses to immune checkpoint inhibitors [140,147].

The interaction between cancer cells and the surrounding stromal compartment is partly mediated by EVs, which contribute to the remodeling of the tumor microenvironment. Cancer cell-derived EVs have been shown to drive the conversion of fibroblasts into cancer-associated fibroblasts (CAFs), reprogram endothelial cells to support angiogenesis, and polarize macrophages and myeloid-derived suppressor cells toward immunosuppressive phenotypes. In turn, CAF-derived EVs reinforce tumor progression by transferring oncogenic RNAs, signaling proteins, and other bioactive molecules back to cancer cells, establishing a reciprocal communication circuit that may sustain malignant growth and therapy resistance. Additionally, EVs have been implicated in multiple steps of the metastatic cascade, including invadopodia formation, matrix remodeling, and organotropism through the delivery of integrins and proteolytic enzymes [148,149].

Notably, EVs are not only agents of malignant communication but also represent valuable tools for clinical applications. Their cargo can be leveraged for minimally invasive diagnostics via liquid biopsy, potentially providing insights into tumor type, stage, mutation profile, and treatment response [150].

Consistent with their immunosuppressive role, particularly through PD-L1-mediated T cell inhibition [140,151], EV-associated PD-L1 has been proposed as a predictive biomarker of therapeutic efficacy in immunotherapy. Circulating EV-bound PD-L1 levels may outperform tissue biopsy for monitoring LC progression, although this remains to be validated in clinical settings [151]. In addition, the inherent biocompatibility and modifiability of EVs are being explored as possible platforms for therapeutic strategies, including EV-based drug delivery systems and engineered vesicles with antitumor or immunomodulatory payloads [152].

In summary, EVs represent a promising and rapidly evolving area of research in tumor communication. However, several proposed functions, particularly those related to systemic dissemination and clinical applications, remain to be fully validated and should be interpreted with caution. A deeper understanding of EV-mediated communication pathways is crucial for advancing cancer biology and may lead to new approaches to biomarker development, enabling early diagnosis and personalized therapy.

### 6.3. EV Impact on Tumor Microenvironment and Metastasis

Among EV subtypes, exosomes are increasingly recognized as playing an important role in LC progression. For instance, Wang et al. [153] performed a proteomic analysis of adenocarcinoma-derived exosomes, identifying proteins enriched in the tumor microenvironment and implicated in metastatic processes, including DEAD-box RNA helicase 18 (DDX18), DnaJ heat shock protein family (Hsp 40) member A3 (DNAJA3), platelet activating factor acetylhydrolase 1b catalytic subunit 3 (PAFAH1B3), BAG cochaperone 6 (BAG6), and carbamoyl-phosphate synthetase 2 (CAD). Among these, PAFAH1B3 was uniquely associated with disease-free survival (DFS) and was shown to promote LC cell invasion and migration via exosomal pathways. Consistent with these findings, in silico analyses confirmed its correlation with poor patient outcomes, supporting its potential as a prognostic biomarker and therapeutic target. Overall, these findings suggest how exosomal cargo may contribute to enhance invasion and migration capabilities, thereby driving metastatic progression [149].

Beyond cancer cells themselves, EVs have been strongly shown to influence the surrounding tumor microenvironment by altering the phenotype of non-malignant cell types, such as fibroblasts and macrophages. Upon exosomal cargo uptake, these cells can be reprogrammed toward a cancer-associated fibroblast or pro-inflammatory phenotype, respectively. This phenotypic shift has been linked to specific signaling cascades, most notably the AKT pathway, triggered by internalized EV components, and it has been associated with downstream cellular processes, including autophagy, mitophagy, and ROS metabolism [148,153]. In turn, components of the TME may influence cancer progression by releasing EV-encapsulated miRNAs, as reported in NSCLC [154]. This supports a bidirectional interplay in which tumor-associated stromal cells can also modulate cancer cell behavior via EV-mediated miRNA transfer. In sum, these findings support the concept of a dynamic, reciprocal EV-mediated crosstalk between tumor cells and their microenvironment, which reinforces the acquisition and maintenance of the malignant phenotype.

Finally, a growing body of evidence suggests that EVs released by LC cells promote cell migration, invasion, and metastasis by transferring non-coding RNAs. These include, for example, lncRNAs such as ENST00000440028/MSL3P1, lnc-MLETA1, and lnc-MMP2-2 [155,156,157], circular RNAs (circRNAs), such as circCCDC134 [157], and miRNAs such as miR-210-3p [158]. These EV-contained RNAs have been implicated in tumor dissemination and may also contribute to chemoresistance mechanisms, as reported for miR-210-3p and others [159].

### 6.4. Potential Therapeutic Targets Within Extracellular Vesicle-Mediated Pathways

As previously mentioned, several LC hallmarks have been reported to be influenced by the molecular cargoes carried by EVs, which regulate key oncogenic signaling pathways. In detail, growing evidence suggests that EVs directly modulate the PI3K/AKT/mTOR, Wnt/β-catenin, and TGF-β pathways, thereby contributing to tumor cell proliferation, colony formation, angiogenesis, EMT, chemoresistance, and resistance to immunotherapy in NSCLC and other LC subtypes [160,161,162,163,164].

Therapeutically, nanotechnology-based strategies are being explored as potential tools to target EV-mediated pathways. For instance, cell-targeted nanoliposomes that inhibit exosome biogenesis and release from lung cancer cells have been shown to prevent fibroblast differentiation into cancer-associated fibroblasts. Additionally, nanoliposomes targeting key signaling pathways activated in EV-recipient cells within the tumor microenvironment have been reported to enhance antitumor immune responses and may improve immunotherapy efficacy, suggesting their potential as components of multimodal therapeutic strategies [162].

Recent studies have further explored the therapeutic potential of engineered EVs, mainly those derived from stem cells, to reshape the TME. These modified EVs have shown, in experimental settings, the ability to reverse immune checkpoint resistance in NSCLC by inhibiting the PI3K/AKT/mTOR pathway, both in vitro and in vivo, with results comparable to those obtained with nanoliposomes [165]. In other words, these findings reinforce the role of this pathway in EV-mediated oncogenic processes.

Targeting the TGF-β signaling axis via delivery of inhibitory Smads or pharmacological agonists such as naringenin and asiatic acid has also shown potential to suppress tumor growth. These agents act by rebalancing Smad signaling in early-stage tumors and mitigating the protumorigenic effects of both tumor-derived and microenvironmental EVs [124,166]. Similarly, it has been shown that Wnt/β-catenin pathway inhibitors, such as IMU1003, pyrvinium pamoate, and fibulin-5, interfere with EV-mediated oncogenic signaling and reduce tumor progression in LC models [167,168,169,170,171].

Overall, while these approaches highlight the therapeutic potential of targeting EV-mediated pathways, most evidence currently derives from preclinical studies, and their clinical applicability remains to be established.

## 7. MNP-EV Signaling and Lung Cancer: Challenges, Therapeutic Perspectives, and Future Directions

The potential role of MNPs in LC remains an emerging and incompletely understood area of research. While experimental studies suggest that MNP exposure may induce biological processes such as chronic inflammation, oxidative stress, and DNA damage, the relevance of these findings to human disease remains uncertain.

Our analysis revealed a rapid increase in publications on the effects of MNP exposure on human health over the last 5 years; however, studies evaluating the impact of MNP-EV interactions on human health remain scarce. Specifically, experimental evidence suggests that MNP exposure may alter EV biogenesis and molecular cargo in epithelial, immune, and stromal cells.

These MNP-modified EVs may carry oncogenic mediators, such as miRNAs, proteins, and lipids, that have been implicated in EMT, angiogenesis, immune evasion, and metastatic potential, thereby influencing cellular processes associated with remodeling of the tumor microenvironment. Altogether, this evidence supports the idea that, unlike EVs involved in physiological or tumor-derived communication, MNP-induced EVs may reflect a stress-induced phenotype, characterized by altered cargo composition and release dynamics associated with environmental exposure. Importantly, the functional consequences of these alterations remain context-dependent, and MNP-induced EVs may contribute to both detrimental, such as pro-inflammatory or tumor-promoting processes and adaptive or protective cellular responses.

**Figure 5:**
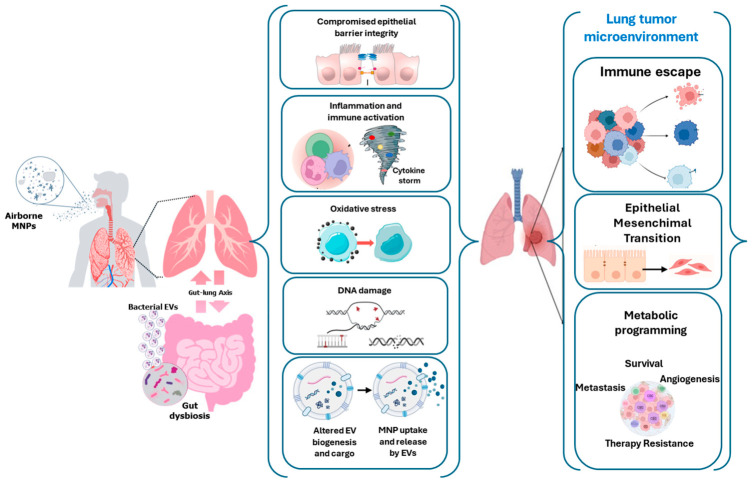
Inhalation exposure to micro- and nanoplastics (MNPs) may contribute to the development of lung cancer by altering extracellular vesicle (EV)-mediated communication. MNPs induce chronic inflammation, oxidative stress, and DNA damage, and modify the biogenesis and molecular content of EVs released by epithelial, immune, and stromal cells. These altered EVs can carry oncogenic mediators (miRNAs, proteins, lipids) that promote epithelial–mesenchymal transition (EMT), angiogenesis, immune evasion, and metastasis, reshaping the tumor microenvironment. Moreover, ingested MNPs may promote intestinal dysbiosis and loss of epithelial integrity, allowing the passage of bacterial (EVs) that, in turn, contribute to inflammation and tumor progression via the gut–lung axis.

EV-mediated communication may extend beyond the lungs and has been hypothesized to promote systemic inflammation and to facilitate distant metastatic processes via the GLA. Intestinal dysbiosis, exacerbated by ingested MNPs, can disrupt epithelial tight junctions, allowing the translocation of bacteria and their EVs into the circulation. These microbial EVs, rich in bioactive molecules, may influence inflammatory and immune processes and potentially affect tumor-related processes, although their contribution to tumor-related mechanisms remains unclear (Figure 5).

However, several mechanistic and methodological challenges remain unresolved. Importantly, the lack of shared standardized protocols for detecting MNPs in biological matrices and characterizing EV subpopulations hinders reproducibility and limits reliable cross-study comparisons. The molecular pathways linking MNP exposure to dysregulated EV biogenesis and function remain incompletely understood, especially under chronic, low-dose conditions representative of real-world conditions. In addition, the discrepancy between experimental exposure levels to MNPs and real-world scenarios represents a critical issue in interpreting the potential health effects of MNPs. Many in vitro and in vivo studies use substantially higher concentrations than those estimated in environmental or human exposure settings. Concentration– and dose–response relationships for MNP exposure remain poorly characterized, and there is currently little information on chronic, low-dose exposure, which more closely reflects real-life conditions. Thus, existing experimental studies, which provide important insights into potential toxicity mechanisms, should be interpreted primarily as evidence of potential hazard rather than as direct indicators of risk to human health. Importantly, these mechanisms may provide a conceptual basis for exploring how MNP exposure may influence LC through EV-mediated signaling.

From a clinical perspective, deciphering MNP-EV signaling may represent a potential area of investigation for developing novel biomarkers and personalized therapeutic strategies for LC. The analysis of EV composition may lead to the development of minimally invasive biomarkers capable of (i) potentially predicting tumor-specific molecular alterations, (ii) enabling early cancer diagnosis, and (iii) supporting disease-state classification and patient stratification. Notably, these biomarkers may also serve as sentinels of MNP exposure, thus supporting preventive strategies in environmental health policies.

Therapeutically, targeting EV-mediated intercellular communication or engineering EVs as biocompatible delivery systems for antitumor agents could be explored as a potential strategy to attenuate MNP-driven carcinogenic signaling with reduced systemic toxicity. This dual role of EVs highlights their potential to bridge environmental exposure research and cancer treatment. However, the development of this potential requires integrated, multidisciplinary approaches that combine exposomic and multi-omic analyses (EV-omics, metabolomics, lipidomics) to correlate exposure to MNPs with molecular and phenotypic alterations detectable in EVs. Such systemic integration could significantly enhance our ability to diagnose, monitor, and treat environmentally driven LC, thereby bridging the gap between environmental toxicology and individualized cancer therapy.

From an environmental and atmospheric perspective, future research should also focus on the transport, transformation, and deposition mechanisms of airborne MNPs, as these processes ultimately influence the real extent of human exposure. Integrating atmospheric physics and climate modeling with toxicological and molecular analyses could yield a more comprehensive understanding of how MNPs interact with both the atmosphere and biological systems.

This interdisciplinary framework would help bridge the current gap between environmental exposure science and biomedical research, supporting a holistic assessment of MNP-related health risks.

## Figures and Tables

**Table 1:** Comparison of the main techniques used for extracellular vesicle (EV) isolation, in terms of efficiency, processing time, and instrumentation.

Method	Time (hours)	Equipment	Recovery Rate	References
Ultracentrifugation	3	Ultracentrifuge	5–25%	[54,56,57,58]
Density gradient centrifugation	3	Ultracentrifuge	60%	[54,56,59]
Polymer-based precipitation	2	Centrifuge	50%	[56,60]
Size exclusion chromatography (SEC)	3	Chromatograph	70%	[54,56,61]
Immunoaffinity capture	1.5	None	70%	[54,56,62]
Microfluidic technologies	1	Microfluidic device	80%	[54,56,63]
Membrane affinity method	0.5	Centrifuge	Not specified	[64,65]

## Data Availability

No new data were created or analyzed in this study. Data sharing is not applicable to this article.

## Abbreviations

The following abbreviations are used in this manuscript:

MPs	Microplastics
NPs	Nanoplastics
MNPs	micro- and nanoplastics
LC	Lung cancer
EVs	Extracellular vesicles
GM	Gut microbiome
PE	Polyethylene
PP	Polypropylene
PS	Polystyrene
PVC	Polyvinyl chloride
PET	Polyethylene terephthalate
HDPE	High-density polyethylene
LDPE	Low-density polyethylene
NSCLC	Non-small cell lung cancer
IL	Interleukin
ROS	Reactive oxygen species
VEGF	Vascular endothelial growth factor
WHO	World Health Organization
MVBs	Multivesicular bodies
ILVs	Intraluminal vesicles
TEM	Transmission electron microscopy
cryo-EM	cryo-electron microscopy
AFM	Atomic force microscopy
NTA	Nanoparticle tracking analysis
DLS	Dynamic light scattering
TRPS	Tunable resistive pulse sensing
MSC	Mesenchymal stem cell
ER	Endoplasmic reticulum
CRC	Colorectal cancer
TX	Thromboxane
PG	Prostaglandin
12S-HETE	12S-Hydroxyeicosatetraenoic acid
GLA	Gut–lung axis
LM	Lung microbiota
PRRs	Pattern recognition receptors
ECs	Endothelial cells
TNF	Tumor necrosis factor
LPS	Lipopolysaccharide
ARDS	Acute respiratory distress syndrome
COPD	Chronic obstructive pulmonary disease
TME	Tumor microenvironment
CAFs	Cancer-associated fibroblasts
EMT	Epithelial–mesenchymal transition
